# *In silico* Analysis and Experimental Validation of Lignan Extracts from *Kadsura longipedunculata* for Potential 5-HT1AR Agonists

**DOI:** 10.1371/journal.pone.0130055

**Published:** 2015-06-15

**Authors:** Yaxin Zheng, Jiming Wu, Xuesong Feng, Ying Jia, Jian Huang, Zhihui Hao, Songyan Zhao, Jinhui Wang

**Affiliations:** 1 Key Laboratory of Structure-Based Drug Design and Discovery of Ministry of Education, Shenyang Pharmaceutical University, Shenyang, 110016, China; 2 School of Pharmacy, China Medical University, Shenyang, 110122, China; 3 School of Traditional Chinese Materia Medica, Shenyang Pharmaceutical University, Shenyang, 110016, China; 4 Animal Center, Shenyang Pharmaceutical University, Shenyang, 110016, China; 5 School of Life Science, Shenyang Pharmaceutical University, Shenyang, 110016, China; University of Edinburgh, UNITED KINGDOM

## Abstract

**Objectives:**

*Kadsura longipedunculata* (KL) has been widely used for the treatment of insomnia in traditional Chinese medicine. The aim of this study was to explore the mechanism of the sedative and hypnotic effects of KL.

**Materials and Methods:**

The content of KL was evaluated by HPLC-TOF-MS, and a potential target was found and used to construct its 3D structure to screen for potential ligands among the compounds in KL by using bioinformatics analysis, including similarity ensemble approach (SEA) docking, homology modeling, molecular docking and ligand-based pharmacophore. The PCPA-induced insomnia rat model was then applied to confirm the potential targets related to the sedative effects of KL by performing the forced swimming test (FST), the tail suspension test (TST) and the measurement of target-related proteins using western blotting and immunofluorescence.

**Results:**

Bioinformatics analysis showed that most of lignan compounds in KL were optimal ligands for the 5-HT1A receptor (5-HT1AR), and they were found to be potential targets related to sedative effects; the main lignan content of KL extracts was characterized by HPLC-TOF-MS, with 7 proposed lignans detected. Administration of KL could significantly reduce FST and TST immobility time in the PCPA-induced 5HT-depleted insomnia rat model. The expressions of proteins related to the 5-HT1AR pathway were regulated by extracts of KL in a concentration-dependent manner, indicating that extracts of KL had 5-HT1AR agonist-like effects.

**Conclusion:**

*In silico* analysis and experimental validation together demonstrated that lignan extracts from KL can target 5-HT1AR in insomniac rats, which could shed light on its use as a potential 5-HT1AR agonist drug.

## Introduction

It has been reported that an estimated 10–15% of the adult population meets the criteria for diagnosis of an insomnia disorder. The current drugs for the clinical treatment of insomnia mainly include benzodiazepine hypnotics and zopiclone drugs [1]. However, these sedative drugs are associated with serious side effects, including impaired coordination, drowsiness and development of tolerance [2]. Therefore, a number of trials continue to seek safer medicines with fewer side effects.

*Kadsura longipedunculata* Finet et Gagnep (Nanwuweizi), which is a type of traditional Chinese medicine, is widely distributed in the southern part of China [3,4]. *Kadsura longipedunculata* (KL) has been used for the treatment of a variety of diseases, including tumors, hepatitis [5,6], inflammation [7], immunosuppression [8], and insomnia [9,10], for thousands of years. Its therapeutic effects on insomnia are highly related to the lignans, which are the main constituents of Kadsura [6,11–13]. Despite its definite curative effects on insomnia, only a few studies have aimed at revealing the sedative mechanism of KL, and the sedative mechanism of KL remains unclear and requires further careful evaluation. To our knowledge, only Zaugg’s study suggested that the sedative effect might be induced by potentiating the gamma-aminobutyric acid (GABA) response through the positive modulation of GABAA receptors [9].

The advent of SEA docking provides us with a good opportunity to seek relevant targets based on the structures of compounds, and the 3D structures of protein targets can be constructed in turn to screen for potential target compounds. In the present study, a potential target related to sedative effects was found and was used to construct its 3D structure to screen for potential ligands among the compounds in KL using bioinformatics analysis, including similarity ensemble approach docking (SEA Dock), homology modeling, molecular docking and ligand-based pharmacophore. The PCPA-induced insomnia rat model was then applied to confirm the potential target relative to the sedative effects of KL by performing the forced swimming test (FST), the tail suspension test (TST) and the measurement of target-related proteins.

## Materials and Methods

### Materials

Fruits of *Kadsura longipedunculata* were purchased from Shenyang, Liaoling Province, People’s Republic of China, in May 2013 and were identified by Professor Jimin Xu (National Institutes for Food and Drug Control). Then, the voucher specimen (No. 20130511) was deposited in the Research Department of Natural Medicine, Shenyang Pharmaceutical University. Anti-5-HT1AR antibody (catalog no. 85615, Abcam, UK), anti-nNOS antibody (catalog no. 4231, Cell Signaling Technology, USA), anti-CREB antibody (catalog no. 9197, Cell Signaling Technology, USA), phospho-CREB (Ser133) antibody (catalog no. 9198, Cell Signaling Technology, USA), anti-PKA antibody (catalog no. 5842, Cell Signaling Technology, USA), phospho-PKA (Thr197) antibody (catalog no. 5661, Cell Signaling Technology, USA), 4-chloro-DL-phenylalanine (PCPA, catalog no. C6506, Sigma, USA) were acquired. An NO kit was obtained from Nanjing Jiancheng Bioengineering Institute (catalog no. 20141017, Jiangshu, China).

### Predictive targets for sedative and hypnotic effects

Target proteins of the reported compounds of *Kadsura longipedunculata* were obtained using the similarity ensemble approach (SEA) (http://sea.bkslab.org/), according to the method described by Wang et al [14]. To search for data from SEA docking, we developed an assistance tool to simplify the process. Copying and pasting such target information can be laborious, and the assistant tool was able to view the source code of the results page and test whether the content matched the pattern set. If the match was successful, the program copied it into a buffer before filing it. With the help of this tool, the task was reduced to inputting a compound and clicking a button. The functions of target proteins were screened on Universal Protein Resource (UNIPROT) (http://www.uniprot.org).

### Homology modeling and molecular docking

The amino acid sequence of 5-HT1AR was downloaded from the UniProt database (accession code: P08908, 5HT1A_HUMAN). Then, the three-dimensional (3D) structure of 5-HT1AR was constructed using the SWISS-MODEL server and gaining the best template (PDBID: 4IAQ). SWISS-MODEL (http://swissmodel.expasy.org), a server for 3D automated comparative modeling of protein structures, has been widely used as a free Web-based automated modeling facility [15]. Some studies have shown that the active state of 3SN6 could also be chosen as a modeling template to construct the key site of 5-HT1AR [16]. A series of procedures of sequence alignment were performed to evaluate the identity of the target sequence and templates. After obtaining a good fit, the 3D structure was constructed based on the homologous template of 3SN6. Hydrogens were added, and then the stripped protein structures were optimized using the Discovery Studio (DS) (Accelrys) software package, version 3.5. The 3D 5-HT1AR protein was then subjected to interacting with the candidate compounds and with ten 5-HT1AR agonists. Before the calculations, the compounds were first optimized for energy under MMFF force fields, and the chemical structures of the candidate compounds were drawn using ChemDraw software, version 12.0. Finally, “-CDOCKER INTERACTION ENERGY” was selected as the major standard for addressing the generated conformations, and “-CDOCKER ENERGY” was selected as a supplementary evaluation over all of the molecular docking procedures.

### Pharmacophore analysis

Three-dimensional pharmacophore models have been widely used to identify the described binding modes and for *in-silico* screening of efficient compounds from the database [17]. In this study, some full agonists chosen as training sets were used to establish a ligand-based pharmacophore with a “*flexible*” fitting method, and the other full agonists acted as test sets and were used to verify all of the returned “hypotheses” [18]. Finally, the most promising models were selected for further evaluation.

### Preparation for enriched lignan extracts of KL

Smashed *Kadsura longipedunculata* fruits were extracted with MeOH (1:10) by supersonic extraction (30 min × 3). The combined MeOH extracts were concentrated in a vacuum at 50°C to generate crude extracts. Then, the extracts were kept in sealed vials at 4°C for further study. The crude extracts were rich in lignans as a result of previous studies [19,20].

### HPLC-TOF-MS analysis

The chromatography analysis was performed on a Waters 2690 Separations Module HPLC system connected to a Waters LCT Premier XE time-of-flight mass spectrometer (Waters, USA). Chromatography was performed using a SunFire C18 column (4.6 × 150 mm, 5 μm), and the column temperature was maintained at 30°C. The mobile phase (delivered at 1 mL/min) consisted of solvent A (H_2_O) and solvent B (acetonitrile), using the following gradient elution procedure: 0–10 min, 38–44% B; 10–35 min, 44–60% B; 35–37 min, 60–47% B; and 37–78 min, 47–85% B. The mass spectrometer was optimized in +V mode, the source temperature was set at 100°C with a cone gas flow of 20 L/h, a desolvation gas temperature of 170°C and a desolvation gas flow of 450 L/h, the capillary voltage was set at 1200 V, the sample cone voltage was set at 100 V, and the extraction cone voltage was set at 100 V. The data were processed by the Masslynx software, version 4.1.

### Animals

Specific pathogen-free male and female Sprague-Dawley (SD) rats, aged 6–8 weeks old and weighing 180–200 g, were obtained from Changsheng Biotechnology Co., Ltd. (Certificate No. SCXK [Liao] 2010–0001, Benxi, China). Animal welfare and the experimental procedures were in accordance with the Ethical Regulations on the Care and Use of Laboratory Animals of Shenyang Pharmaceutical University and were approved by the Animal Experimental Ethics Committee for all animal experiments.

### Acute oral toxicity

The experimental protocol was approved by the Animal Experimental Ethics Committee of Shenyang Pharmaceutical University (Permit Number: SYPU-IACUC-C2015-0514-005). Twenty-four male and female SD rats (n = 6) were randomly divided into a control group and three dose level groups (low, medium, high groups administered 5, 10, and 20 g KL/kg body weight/day, respectively). KL was administered once by oral gavage at 10 mL/kg body weight, and the control group was given the same volume of saline. The general behavior, body weight and condition of the rats were monitored at 1, 2, 4 and 6 h and then once per day for 14 days [21].

### Establishment of PCPA-induced insomnia rat model

This animal experiment was also approved by the Animal Experimental Ethics Committee of Shenyang Pharmaceutical University (Permit Number: SYPU-IACUC-C2015-0514-006). Male SD rats (12 rats per group) were assigned to one of six groups. Three groups received oral KL (4 g/kg, 8 g/kg, 12 g/kg) continuously for 7 days, followed by i.p. PCPA (300 mg/kg) at day 4 for three days. Two groups received oral saline (control group) and buspirone (positive group), followed by i.p. PCPA (300 mg/kg) at day 4 for three days. Saline was given to one group as a blank group. The dose of KL was converted to the quality of the original plants. The preparing training for the FST and TST were undertaken on day 3 after treatment. Finally, the FST and TST immobile times were measured on day 6. After 7 days of treatment, the rats were anesthetized and sacrificed by cervical dislocation, and the brains were removed and placed immediately on ice.

### Assays with the forced swim test (FST) and tail suspension test (TST)

The sedative and hypnotic effects of KL on PCPA-induced insomniac rats were assessed by FST and TST assays, as previously described [22]. The rats were placed in individual, clear glass cylinders (40 cm tall × 18 cm diameter) containing 25°C water, approximately 23 cm deep, to prevent their tails from touching the bottom [23]. Immobility was manually recorded over the total 6 min testing period. Rats were considered to be immobile when they made the small movements necessary to keep their heads above the water surface. In the tail suspension test, the rats were individually suspended on the edge of a shelf approximately 50 cm above a table by adhesive placed on the tail [24]. The time spent immobile during a 6-min testing period was recorded. All of the tests were conducted in quiet room to avoid unnecessarily disturbance to the rats.

### Western blotting analysis

For western blotting analysis, the rats were sacrificed by cervical dislocation under deep chloral hydrate anesthesia on the 7th day after treatment with KL extracts. The brains of the rats were isolated on ice immediately, and the hippocampus of the brain was separated at the same time. The hippocampus was lysed in ice-cold lysis buffer with 0.01 mM phenylmethanesulfonyl fluoride (PMSF) and then was smashed with a homogenizer in an ice bath. The samples were then centrifuged at 10000 rpm for 10 min at 4°C. The supernatants were stored at -80°C for further use. The protein concentrations of the brain were measured with a BCA protein assay kit, following the manufacturer’s instructions. All of the samples were boiled for 5 min with SDS-PAGE loading buffer for protein denaturation. Before western blotting, 10% and 8% SDS-polyacrylamide gels were prepared. The equivalent amount of protein (50 μg) was separated on SDS-polyacrylamide gel for 2 hours and then was transferred onto a polyvinylidene difluoride membrane. The protein membranes were blocked with 5% bovine serum albumin or 5% skim milk for 2 h at room temperature and were subsequently incubated with the primary monoclonal antibody overnight at 4°C. The primary antibodies against 5-HT1AR, PKA, p-PKA, CREB, p-CREB and nNOS were diluted to 1:1000 and β-actin to 1:10000. All of the membranes were washed with Tris-buffered saline supplemented with 0.1% Tween 20 (TBST) (3 × 10 min) and were incubated with the secondary antibody for 2 h at room temperature. The membranes were then washed with TBST three times (3 × 10 min). The signals were measured using an ECL western blotting detection system (Beyotime Institute of Biotechnology, Haimen, China). Relative intensities were normalized using β-actin as an internal standard.

### Estimation of NO

The hippocampi of the rats were dissected at 4°C and were homogenized in ice-cold saline. The homogenates were centrifuged at 2500 rpm for 10 min at 4°C, and the supernatants were removed for further analysis. The NO level in the hippocampus tissue was measured using a Nitric Oxide Assay kit, according to the protocol of the kit. Absorbance was read at 550 nm using a microplate reader (Thermo Fisher Scientific, USA). The amount of NO in the hippocampus was measured using a standard sodium nitrite solution and was expressed in μM/g of protein, and the concentration of protein was tested with a BCA protein assay kit.

### Immunofluorescence

For immunofluorescence analysis, the rats were anaesthetized using chloral hydrate and then were perfused with 150 mL of saline solution and 150–200 mL of 4% paraformaldehyde in 0.1 M phosphate buffer (PB). Then, the brains of the rats were isolated and fixed in 4% paraformaldehyde for 2–3 h. The rat brain sections were cut on a freezing microtome at a thickness of 16 μm and were washed with phosphate-buffered saline (PBS) and pre-incubated in 5% horse serum. Subsequently, the sections were incubated with 5-HT1AR antibody for 24 h at 4°C, washed with PBS and incubated with the secondary antibody and neuro tracer for 2 h at room temperature while protected from light [25]. The sections were examined on a Nikon C2 plus confocal laser-scanning microscope (Nikon, Japan).

### Statistical analysis

The data are expressed as the means ± SDs. The differences were analyzed by one-way analysis of variance (ANOVA) using SPSS software, version 16.0. Our data were considered significant at different levels when *P* < 0.05, *P* < 0.01, and *P* < 0.001.

## Results

### Identification of 5-HT1AR as the target of KL

To identify the targets related to the sedative effects of KL, 131 compounds (55 lignans, 14 terpenes, 37 phenylpropanoids, 12 phenolic acids, 3 sterides and 10 others) were collected with Scifinder (https://scifinder.cas.org/). These compounds were then input into SEA to obtain targets related to sedative effects, and the 5-HT1AR protein was found to be the optimal target related to neurological diseases in brain because 16 compounds (12 lignans) in KL were found to target 5-HT1AR.

### Molecular modeling and docking of 5-HT1AR

The CDOCKER protocol in Discovery Studio software can be classified as a kind of molecular docking tool, which is distinguished by its precise binding mode between ligands and receptors. The simulation algorithm in this protocol simulates annealing. Combined with the grid-based conformational search method and the most appropriate conformation of the ligand, the ligand’s most favorable binding mode with receptors in the active site can be chosen effectively. The ligand-receptor interactions between reported full agonists and the 5-HT1AR protein were investigated before some other interactions between the proved structures of the compounds in KL and 5-HT1AR protein were explored. As a result, H-bond interactions between most agonists and Asn386 in the protein active site could be found. Furthermore, there also existed other agonists that could form H-bonds with Ile116. Noticeably, the hydrophobic parts of most agonists could embed in the hydrophobic pocket formed by the lipophilic residues of Phe112, Ile113 and Asp116. Based on the information above, the 5-HT1AR protein amino acids Asn386 and Ile116, as well as the hydrophobic pocket formed by Phe112, Ile113 and Asp116, were recognized as critical factors on the agonist effects of KL.

**Fig 1:**
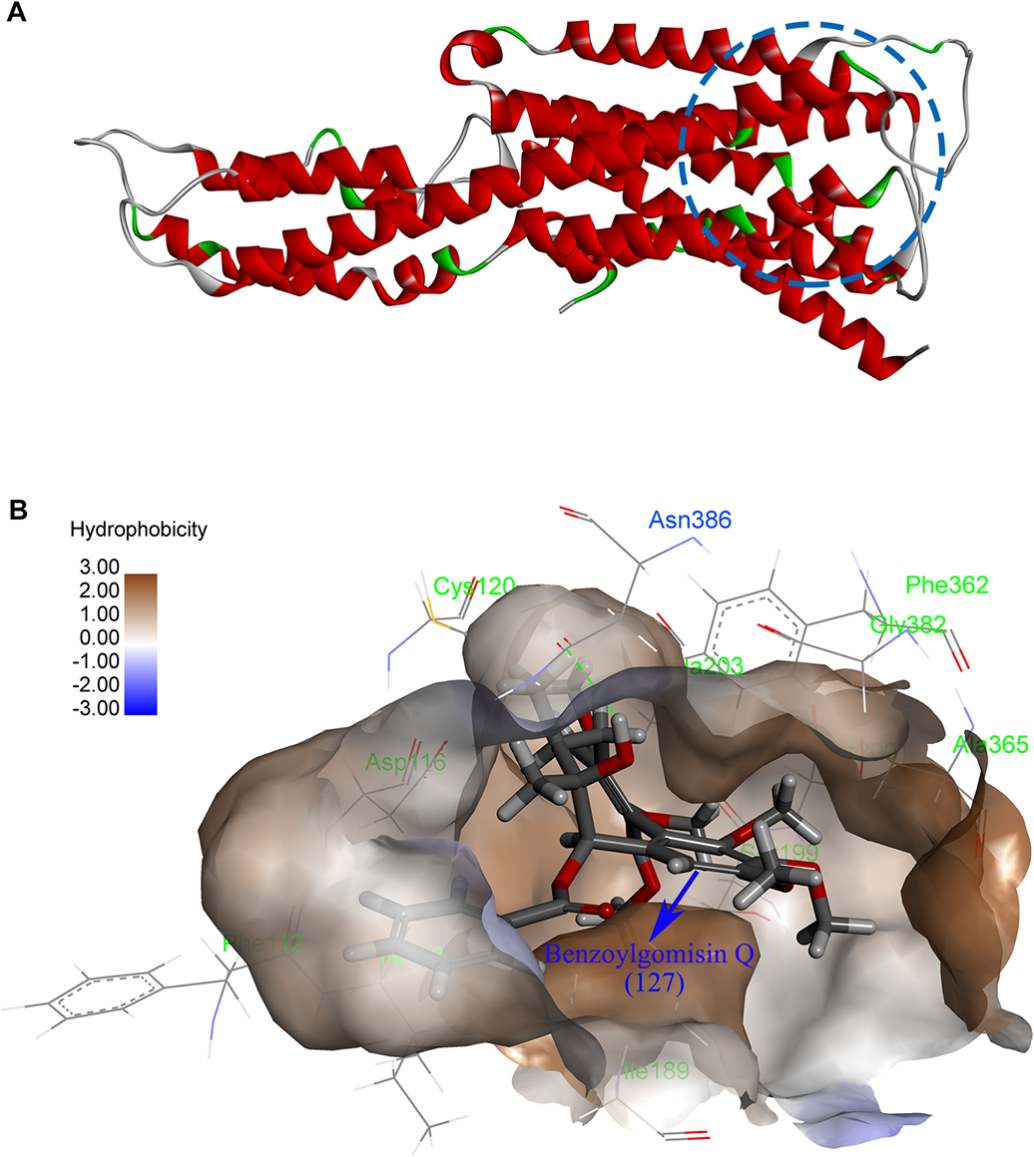
The structure of the 5-HT1AR protein. (A) Homology model of the 5-HT1AR in drawn representation. (B) The interaction of compound 127 with the 5-HT1AR.

To predict the potential 5-HT1AR agonists in *Kadsura longipedunculata* Finet et Gagnep, CDOCKER module-based molecular docking and “-CDOCKER INTERACTION ENERGY”-based criticism of the stability of ligand-receptor complexes were employed to study the interactions between the known compounds in KL and 5-HT1AR protein. To obtain accurate docking results, all of the candidate compounds in KL were limited to a radius of a 5Å active sphere to perform conformational searches. The top five binding modes of the resulting ten complexes with the highest absolute values of docking energies were preserved. Upon the analysis of these docking results, lignans were identified as the major compounds with high “-CDOCKER INTERACTION ENERGY” values. All of these compounds exhibited strongly hydrophobic effects and ideally matched the hydrophobic property of the 5-HT1AR active site (Fig 1A). Furthermore, the lignan compounds **13**, **131** and **127** were well incorporated via H-bond interactions with Asn386 in the structure analysis (Fig 1B), in which these three compounds, which showed satisfactory binding modes with 5H-T1AR, were also contained in the predicted results using the SEA Dock program. In addition, the top five binding modes of the three compounds with 5-HT1AR that could form H-bonds with Asn386 were proved to be the optimal compounds with docking energy and intragroup binding modes. Noticeably, the five top complexes of compound **127** (Fig 1B), which formed H-bond effects with Ile116, were shown to have lower absolute values of docking energy than the binding mode with Asn386, indicating that Asn386 might play a more important role than Ile116 in 5-HT1AR agonism.

### Ligand-based pharmacophore generation and validation

**Fig 2:**
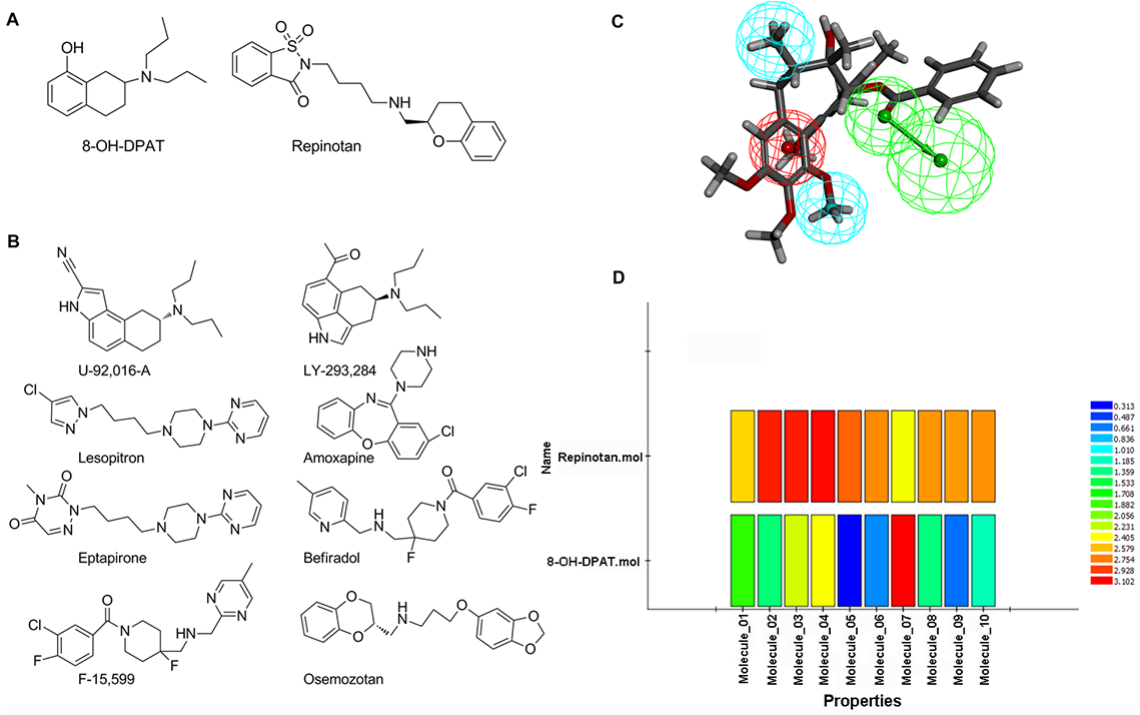
The validation of ligand-based pharmacophore. (A) The structure of text sets. (B) The structure of training sets. (C) Pharmacophore model of 5-HT1AR ligand mapping with compound 127, containing one acceptor center (green), one positive ionizable center (red) and one hydrophobic center (blue). (D) Mapping of two test set compounds onto ligand-based pharmacophores; all mappings were marked with different colors, and high scores referred to good fits.

**Fig 3:**
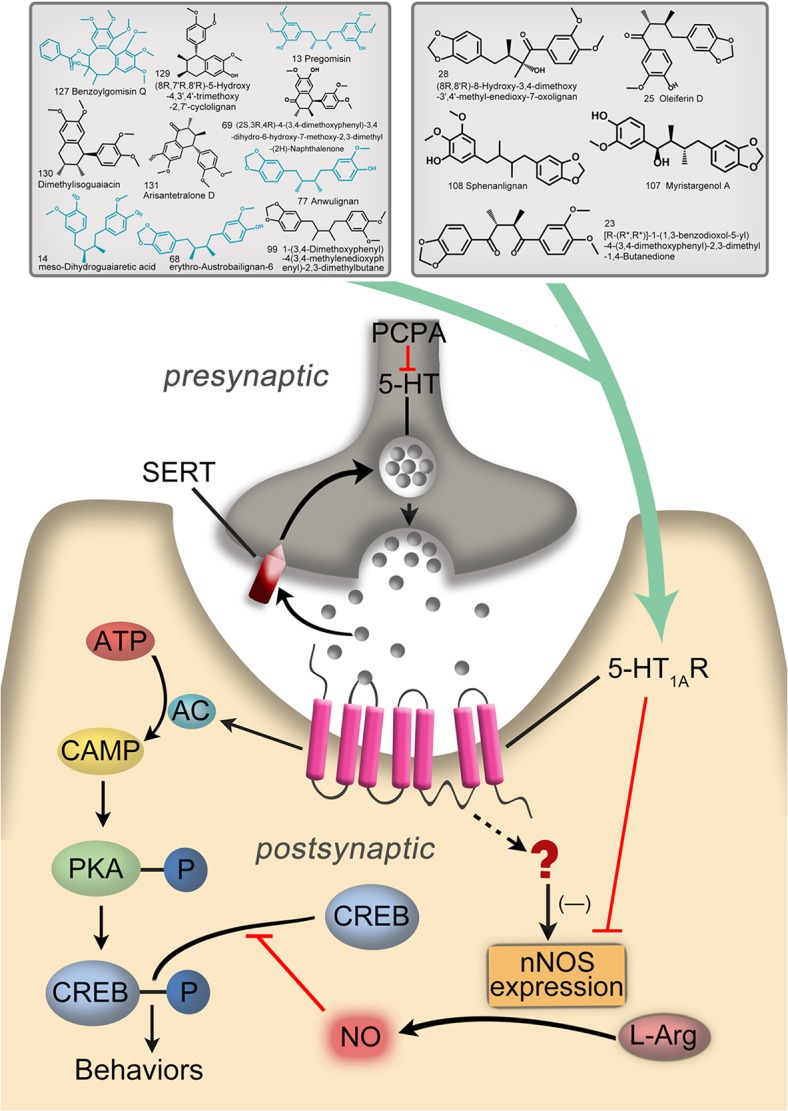
Schematic model of this study.

To validate further the results generated by molecular docking, ten 5-HT1AR full agonists were selected to establish ligand-based pharmacophores by text mining. Combined with the generated pharmacophore models, potential 5-HT1AR agonists among our compounds could be screened. Further verifications were processed regarding whether activity-predicting ability was incorporated in the pharmacophore built herein. Based on the “diverse molecules” systematics in the “Generating Training and Test Data” module, 8-OH-DPAT and repinotan were designated as the test set (Fig 2A), while the remaining eight full agonists were determined as the training set (Fig 2B). Finally, the training set compounds were managed to establish ten ligand-based pharmacophores (Hypo) (Table 1). More insights were gained from these Hypos, and the major pharmacodynamic features that were confirmed included hydrophobic features (H), ring aromatic features (R), H-bond accepters (A), and pos ionizable features (P). To evaluate screening capacity of 5-HT1AR agonists, approximately 10 pharmacophores from the remaining two test set molecules were used to map with ten Hypos. As shown in Fig 2D, repinotan could map well onto all ten of these Hypos, while 8-OH-DPAT exhibited poor performance in mapping with Hypo **5**, **6**, **8**, **9** and **10**. Considering the screening capacity of 5-HT1AR agonists, Hypo **1**, **2**, **3**, **4**, and **7** were preliminarily kept. Further studies showed that the pharmacodynamic features of these Hypos all consisted of PHHA. Considering the scores of the five Hypos and their mappings with the test set compounds, Hypo **4,** which was finally screened and validated, was able to select the potential 5-HT1AR agonists (Fig 2C). Based on this ability, all 131 of our compounds were mapped onto Hypo **4** to screen for possible 5-HT1AR agonism structures. Considering the few compounds containing N elements, the pos ionizable feature (P) would have a negative effect on the screening process. Accordingly, the “Maximum Omitted Features” value in the *Ligand Profiler* protocol was set to -1 before mapping, indicating that only one pharmacophore element was allowed to be mapped incorrectly or not mapped. Under these conditions, most of our compounds were successfully mapped onto Hypo **4**. To obtain more accurate screening results, the low limit of fit value for judging active compounds was set at 2.9, and fifty-six compounds that met the criteria were selected. Compound **127**, which was chosen by both molecular docking and SEA Dock, was also found to map excellently onto the hydrophobic features (H), ring aromatic features (R) and H-bond accepters (A) of Hypo **4**. Because no positive charge was included, it was inappropriate for pos ionizable features (P) mapped with **127**. Nevertheless, it was noticeable that 77% of the screened compounds were lignans, among which 20% of the lignans proved to have the same structures as those of the docking-predicted potential agonists. Subsequently, compounds fulfilling both the molecular docking and pharmacophore screening methods were searched and compared with the structures predicted by SEA Dock. All the same compounds of **13**, **14**, **68**, **77** and **127** were lignans (Fig 3). As shown in Fig 3, ten compounds in the upper left corner were selected by SEA Dock and molecular docking. Compounds marked in blue were mapped onto Hypo **4** ideally; the five compounds in the upper right corner were selected by SEA Dock and Hypo **4**. Therefore, we speculated that the lignan compounds for KL were the potential 5-HT1AR agonists.

**Table 1:** Scores of common feature based pharmacophore hypotheses.

NO.	Features	Score	Direct Hit	Partial Hit	Max Fit
**1**	PHHA	66.338	11111111	00000000	4
**2**	PHHA	63.937	11111111	00000000	4
**3**	PHHA	63.600	11111111	00000000	4
**4**	PHHA	61.168	11111111	00000000	4
**5**	RPH	58.748	11111111	00000000	3
**6**	RPH	58.518	11111111	00000000	3
**7**	PHHA	56.919	11111111	00000000	4
**8**	RPH	55.257	11111111	00000000	3
**9**	RPH	54.820	11111111	00000000	3
**10**	RPH	54.397	11111111	00000000	3

P, pos Ionizable, H, hydrophobic; A, H-bond acceptor; R, ring aromatic.

### Characterization of the constitution of KL by HPLC-TOF-MS

HPLC-QTOF-MS was employed to analyze the crude extracts of KL, and 70 components (30 lignans) were identified by comparing the exact masses and formulae of their MS data by HPLC-MS. Among them, 7 lignans matched well with 5-HT1AR, based on the results of bioinformatics analysis as described above, including arisantetralone D (**131**), (2S,3R,4R)-4-(3,4-dimethoxyphenyl)-3,4-dihydro-6-hydroxy -7-methoxy-2,3-dimethyl-(2H)-naphthalenone (**69**), myristargenol A (**107**), sphenanlignan (**108**), pregomisin (**13**) [26], [R-(R*,R*)]-1-(1,3-benzodioxol-5-yl)-4-(3,4-dimethoxyphenyl)-2,3-dimethyl-1,4-butanedione (**23**), and benzoylgomisin Q (**127**) [27] (Table 2, S1 Fig).

**Table 2:** The retention time (Rt) and MS characteristics of the main detected peaks in KL sample.

Compound No.	Rt	Calculated	[M+H]^+^	Error (ppm)	Formula
**131**	11.394	357.1702	357.1708	1.7	C_21_H_24_O_5_
**69**	12.334	357.1702	357.1704	0.6	C_21_H_24_O_5_
**107**	12.751	345.1702	345.1696	-1.7	C_20_H_24_O_5_
**108**	22.613	359.1858	359.1876	5.0	C_21_H_26_O_5_
**13**	22.760	391.2121	391.2117	-1.0	C_22_H_30_O_6_
**23**	34.936	371.1495	371.1506	3.0	C_21_H_22_O_6_
**127**	50.137	553.2438	553.2433	-0.9	C_31_H_36_O_9_

### Acute oral toxicity study

No mortalities were observed in the animals throughout the test period following oral administration of various doses of KL, and no changes in general behavior, body weight (Table 3) or other physiological activities were observed, compared with the control group. These results indicated that acute exposure to KL did not cause obvious toxic effects, and the LD_50_ value of KL in rats was considered to be greater than 20 g/kg.

**Table 3:** The change of body weight in acute oral toxicity study (n = 6).

Dose (g/kg)	Day 1	Day 5	Day 10	Day 14
**0**	192.9±3.0	216.0±10.9	249.3±14.3	273.8±11.9
**5**	191.8±5.2	216.0±8.3	243.9±2.3	271.8±3.9
**10**	195.2±5.7	210.5±3.9	242.8±7.4	272.8±8.2
**20**	194.0±2.3	212.0±7.4	240.0±3.4	267.4±6.4

### Protective effects of lignan extracts in KL against PCPA-induced rat insomnia

**Fig 4:**
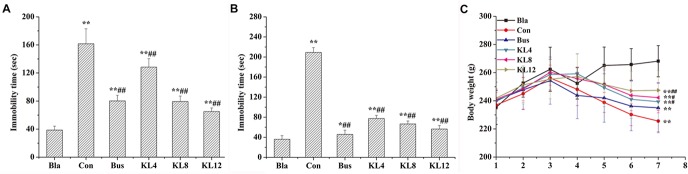
Effects of KL on the TST, FST and daily body weight of PCPA-induced insomniac rats. (A) Immobility time in the TST. (B) Immobility time in the FST. *, **: different from the immobility time of the blank group, *P* < 0.01, *P* < 0.001; #, ##: different from the immobility time of the control group, *P* < 0.01, *P* < 0.001; n = 12, mean ± SD. (C) The effect of KL on daily body weight. **: different from the body weight of the blank group, *P* < 0.001, n = 12, mean ± SD; #, ##: different from the body weight of the control group, *P* < 0.05, *P* < 0.001, n = 12, mean ± SD 1. Bla: blank group, Con: control group, Bus: buspirone group, KL: *Kadsura longipedunculata* group.

The lignans from KL were found to be potential 5-HT1AR agonists, based on the results of extensive bioinformatics analysis as described above. To confirm the results of bioinformatics, the PCPA-induced 5HT-depleting insomnia model was used to study the sedative and hypnotic effects of KL by evaluating the immobility time in the FST and TST, which have been generally considered to be specific behavioral tests for anxiolytic and antidepressant-like effects. Fig 4A and 4B show the effects of KL on the immobility time in the FST and TST. One can see that the administration of KL at all doses could significantly decrease the immobility time in the FST and TST compared to the control group (*P* < 0.001), indicating that the oral administration of KL was effective in producing sedative and hypnotic effects in these behavioral models. The protective effects of KL were highly dependent on the dose, and the high dose (12 g/kg) of KL exhibited excitingly similar effects, compared to the positive control drug (buspirone). In addition, the variations in daily body weight are shown in Fig 4C. One-way ANOVA suggested that continuous treatment with KL produced a significant dose-dependent increase in body weight compared to the control group (*P* < 0.05), whereas buspirone failed to decrease weight loss significantly (P = 0.076), indicating the greater efficacy of KL in reducing weight loss in the PCPA-induced insomnia model, compared to the positive control groups. It is worthwhile to mention that the weight loss at day 4 was due to the FST and TST training on day 3. These results indicated that KL had significant protective effects on PCPA-induced insomniac rats.

**Fig 5:**
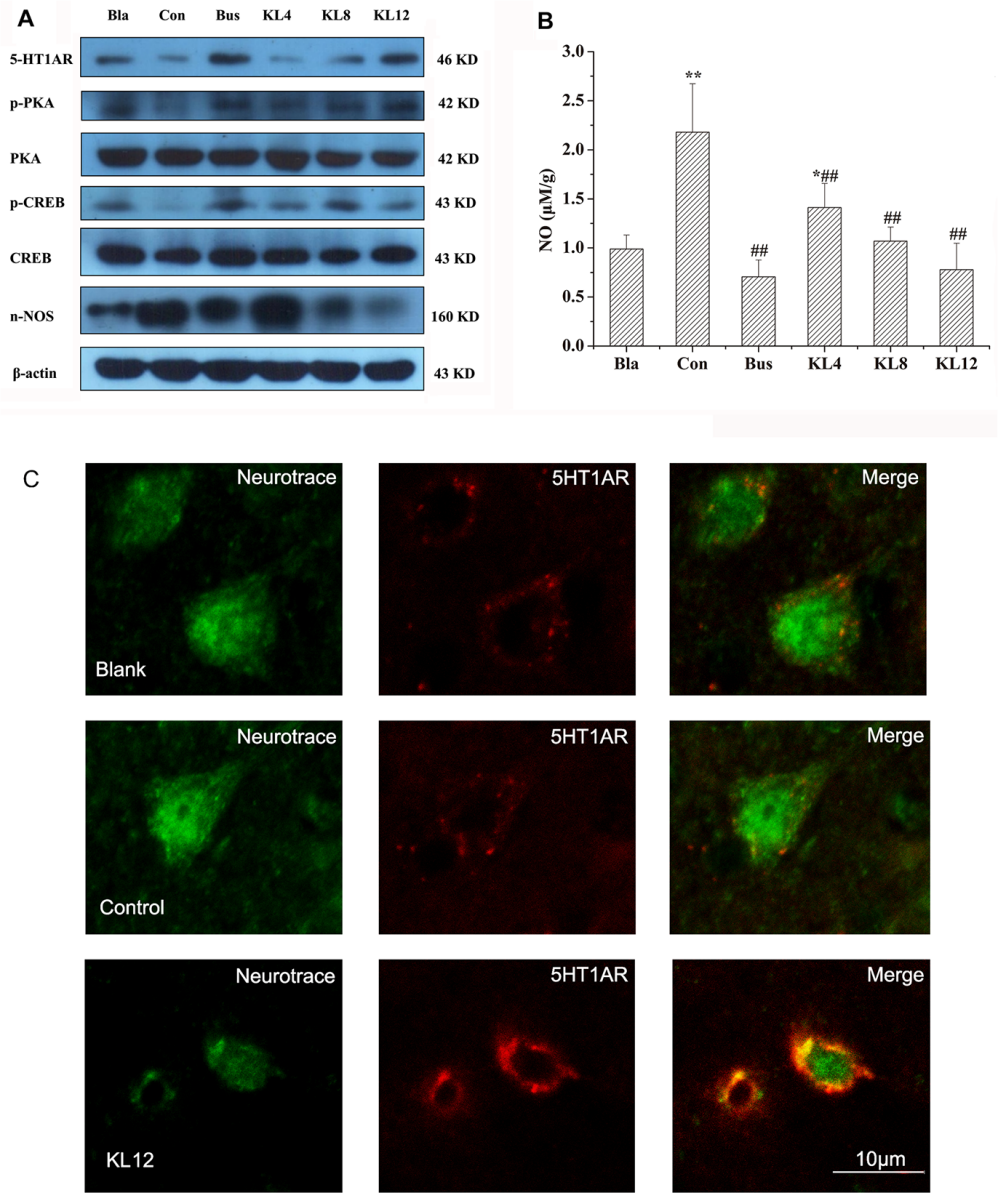
Effects of KL on the NO release and expression of target proteins measured by western blotting and immunofluorescence analysis. (A) Immunoblots of brain hippocampus homogenates with antibodies specific for 5-HT1AR, p-CREB, CREB, p-PKA, PKA, and nNOS. (B) Effects of KL on the release of NO in the hippocampus. *, **: different from the NO content of the blank group, *P* < 0.05, *P* < 0.001; ##: different from the NO content of the control group, *P* < 0.001; n = 12, mean ± SD; Bla: blank group, Con: control group, Bus: buspirone group, KL: *Kadsura longipedunculata* group. (C) Confocal immunofluorescence analysis of rat hippocampus with 5-HT1AR mAb (red) and neuro trace (green). The neuro trace was used to identify neurons.

To study further whether 5-HT1AR was associated with the sedative and hypnotic effects of KL, the changes in proteins related to the 5-HT1AR pathway in the hippocampus were investigated the using western blotting method, because 5-HT1AR is richly expressed in the hippocampus [28,29], where neurogenesis implications are regulated by anxiety-related behaviors [30,31]. Many recent studies have demonstrated that the cAMP (cyclic adenosine monophosphate)/PKA (protein kinase A)/CREB (cAMP-response element binding protein) pathway could be modulated by 5-HT1AR in the hippocampus, resulting in changes in anxiety-related behaviors. On the other hand, 5-HT1AR also selectively exerted negative control on nNOS expression [30], which decreased NO synthesis and subsequently promoted the phosphorylation of CREB. As shown in Fig 5A, the expression of 5-HT1AR in the hippocampi of rats treated with KL was significantly increased in a dose-dependent manner compared to the control group after seven days treatment with KL, and similar expression of 5-HT1AR was observed between the KL group (12 g/kg) and the positive group. The levels of p-PKA and p-CREB protein in the KL groups were upregulated, compared with the control group. Interestingly, the level of nNOS in the hippocampus was sharply decreased, particularly in the high-dose KL group (12 g/kg). Correspondingly, a dose-dependent decrease in NO level in the hippocampus was also noted following the administration of KL, compared to the control group (Fig 5B). Actin was used as a loading control for immunoblotting experiments.

To confirm further the up-regulation of 5-HT1AR in the hippocampus by treatment with KL, immunofluorescence was used to detect the phenotype of 5-HT1AR-positive cells. As Fig 5C shows, co-localization of 5-HT1AR antibody (red fluorescence) and the neuronal marker neuro tracer (green fluorescence) in the rat hippocampus was observed compared to the blank and control groups, indicating up-regulation of 5-HT1AR. This result agreed well with a previous report that 5-HT1AR in PCPA-treated rats was down-regulated, whereas a 5-HT agonist could inhibit the down-regulation, probably due to decreased disruption of 5-HT1AR gene transcription [32]. The results, combined with the results of western blotting, clearly showed that KL administration could upregulate 5-HT1AR in the hippocampus of the rat, just as the 5-HT1AR partial agonist (buspirone) does. Therefore, we reasonably concluded that KL extracts might exhibit a 5-HT1AR agonist-like effect in the PCPA-induced 5HT-depleting insomnia model.

## Discussion

As we known, several drugs targeting pathways have been associated with the treatment of insomnia, such as GABAA agonism, melatonergic agonism, serotonin receptor modulation and orexin receptor antagonism [33]. However, the targeted pathway related to the sedative effects of KL remains unclear. Owing to the development of computation simulation technologies, the in-depth interactions between target proteins and ligands can be more successfully understood [34]. In this study, the compounds in KL were input into SEA Dock to research the potential compound-related targets. Subsequently, 16 compounds in KL matching 5-HT1AR were chosen to construct its 3D structure using homology modeling. Then, all of the compounds in KL were assessed further using molecular docking and ligand-based pharmacophores, and the lignans were found to be potential 5-HT1AR agonists exhibiting sedative effects. In fact, lignans have been identified as active ingredients for the sedative effects of KL in many studies [20,35], but the potential targets of the lignans have remained unclear. This study was the first to identify 5-HT1AR agonist-like effects of lignans in KL using the bioinformatics analysis.

The PCPA-induced insomnia rat model was then used for the verification of the sedative effects of KL extracts. PCPA is serotonin synthesis inhibitor that has been widely used to produce the serotonin-depleting symptoms of insomnia after 3 consecutive days of administration [36]. In our study, all of the insomniac rats were treated with lignan extracts or buspirone (5-HT1AR partial agonist) for 4 days before the peritoneal injection of PCPA. Our results showed that the lignan extracts resulted in significantly decreased immobility time on the FST and TST in PCPA-produced 5-HT-depleted rats, indicating potential sedative effects.

The previously documented studies showed that 5-HT1AR was related to anxiolytic, antidepressant, antipsychotic, neuro-protective and analgesic effects, and it mainly exists in the cortex, striatum, hypothalamus, amygdala and hippocampus [37,38]. To confirm the potential 5-HT1AR agonist-like effects of extracts of KL, the changes in proteins related to the 5-HT1AR pathway in the hippocampus were measured using western blotting and immunofluorescence methods. Our results showed that the administration of KL could significantly upregulate the expression of 5-HT1AR. This result agreed well with previous studies in which serotonergic drugs could significantly impact the number of 5-HT1A receptors expressed in neonatal rat brains [32], and a deficit in 5-HT1AR was concurrent with anxiety-related behavioral changes [39], whereas early developmental overexpression of the 5-HT1AR could decrease anxiety [40]. As downstream proteins of the 5-HT1AR pathway, we found that p-CREB and p-PKA in the hippocampus were upregulated due to the 5-HT1AR agonist-like effects of KL. Similar results have been reported regarding deficits of hippocampal CA1 5-HT1AR being connected to disturbances of cAMP/PKA/CREB signaling [41–43]. Therefore, we reasonably assumed that the administration of lignan extracts might activate the 5-HT1AR protein, subsequently impacting the expression of downstream proteins. In contrast, the effects of hippocampal nNOS and CREB were modulated by activation of the 5-HT1AR [30], and nNOS regulated the quantity of NO, which promoted the phosphorylation of CREB [44]. Therefore, the expression of nNOS and the NO quantity in the hippocampus were also measured, and we found downregulation of nNOS and the suppression of release of NO, further demonstrating that the sedative and hypnotic effects of extracts of KL might arise from the activity of 5-HT1AR agonism.

Combining the results of bioinformatics analysis and *in vivo* studies, we assumed that lignans in KL played roles as the main ligands of the 5-HT1AR. It is well known that a good neurotherapeutic drug should be able to pass the blood-brain barrier (BBB) to reach the brain target site [45]. Many studies have showed that lignans containing typical aromatic rings can cross the BBB [46]. For example, the typical lignan honokiol is able to cross the BBB and the blood-cerebrospinal fluid barrier, and it exerts an anticancer effect [47,48]. Furthermore, six lignans have been detected directly in rat brain tissue after oral administration of lignan extracts [49]. Therefore, the present investigations showed that lignans have the greatest potential for crossing the BBB and modulating the nervous system via a 5-HT1AR agonist-like mechanism.

## Conclusion

The current study demonstrated that the lignans in KL showed potential 5-HT1AR agonist-like effects, based on the results of *in silico* analysis and experimental validation, which could shed light on exploring KL as a potential 5-HT1AR agonist drug.

## Supporting Information

S1 Fig Total ion chromatogram of extracts of KL and extracted ion chromatograms of 7 proposed compounds obtained from LC-TOF-MS analysis. (TIF)
