# Supplementary figures and images for: In silico Analysis and Experimental Validation of Lignan Extracts from Kadsura longipedunculata for Potential 5-HT1AR Agonists
Source: PLoS One. 2015 Jun 15;10(6):e0130055. doi: 10.1371/journal.pone.0130055 (PMC4468062; doi:10.1371/journal.pone.0130055)

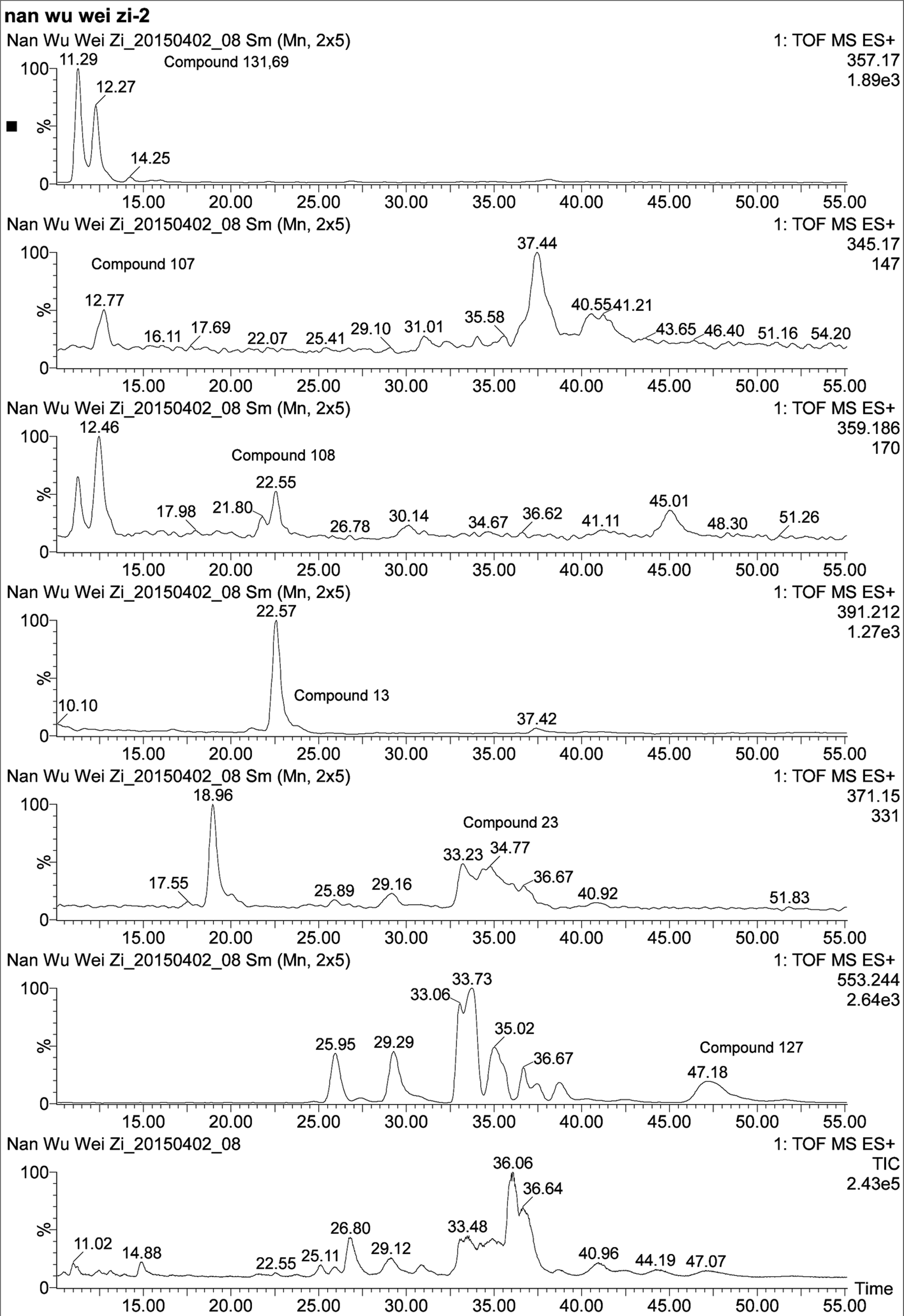

Supplement: S1 Fig — (TIF) [file pone.0130055.s001.tif]
